# How social interactions affect emotional memory accuracy: Evidence from collaborative retrieval and social contagion paradigms

**DOI:** 10.3758/s13421-016-0597-8

**Published:** 2016-02-23

**Authors:** Elizabeth A. Kensinger, Hae-Yoon Choi, Brendan D. Murray, Suparna Rajaram

**Affiliations:** Department of Psychology, Boston College, McGuinn Hall Rm. 300, 140 Commonwealth Avenue., Chestnut Hill, MA 02467 USA; Department of Psychology|, Stony Brook University, Stony Brook, NY USA

**Keywords:** Emotion, Social influences, False memory, Recognition, Valence

## Abstract

In daily life, emotional events are often discussed with others. The influence of these social interactions on the veracity of emotional memories has rarely been investigated. The authors (Choi, Kensinger, & Rajaram *Memory and Cognition*, 41, 403–415, 2013) previously demonstrated that when the categorical relatedness of information is controlled, emotional items are more accurately remembered than neutral items. The present study examined whether emotion would continue to improve the accuracy of memory when individuals discussed the emotional and neutral events with others. Two different paradigms involving social influences were used to investigate this question and compare evidence. In both paradigms, participants studied stimuli that were grouped into conceptual categories of positive (e.g., celebration), negative (e.g., funeral), or neutral (e.g., astronomy) valence. After a 48-hour delay, recognition memory was tested for studied items and categorically related lures. In the first paradigm, recognition accuracy was compared when memory was tested individually or in a collaborative triad. In the second paradigm, recognition accuracy was compared when a prior retrieval session had occurred individually or with a confederate who supplied categorically related lures. In both of these paradigms, emotional stimuli were remembered more accurately than were neutral stimuli, and this pattern was preserved when social interaction occurred. In fact, in the first paradigm, there was a trend for collaboration to increase the beneficial effect of emotion on memory accuracy, and in the second paradigm, emotional lures were significantly less susceptible to the “social contagion” effect. Together, these results demonstrate that emotional memories can be more accurate than nonemotional ones even when events are discussed with others (Experiment 1) and even when that discussion introduces misinformation (Experiment 2).

Individuals tend to feel confident in the durability and accuracy of emotional memories. The term “flashbulb memory,” coined by R. Brown and Kulik (1977), captures the picture-like vividness that individuals often ascribe to their memories of surprising and emotionally arousing events. Empirical data, however, indicate that memories for emotional events are vulnerable to distortion: Individuals often change their reports about how they first learned of an emotional event (e.g., Neisser & Harsch, 1992; Schmolck, Buffalo, & Squire, 2000; Weaver, 1993), and nonpresented emotional items can be falsely endorsed (e.g., Pesta, Murphy, & Sanders, 2001). Although these studies demonstrate convincingly that emotional memories are not immune to distortion, debates have continued about whether emotional content influences the frequency with which memory distortion occurs. Many studies have reported higher rates of false recall and false recognition of emotional items than of neutral ones (e.g., Brainerd, Stein, Silveira, Rohenkohl, & Reyna, 2008; Gallo, Foster, & Johnson, 2009), although in the majority of these studies the conceptual or thematic relatedness of the emotional items was not matched to those of the neutral items (see Gallo et al., 2009, for discussion). We have recently shown (Choi, Kensinger, & Rajaram, 2013) that when this factor is equated across valences, by using categorized study lists, emotionally arousing items are more accurately remembered than are neutral items, and there is no tendency for emotion to enhance false recognition. Palmer and Dodson (2009) reported a related finding: When backward associative strength and word frequency were equated across associate lists that differed in valence, the positive or negative word lists resulted in lower false recall rates than did the neutral words lists. Based on the manipulations within that study (e.g., exclusion and inclusion instructions), they interpreted these effects of emotion as stemming both from the way information was encoded and also to the way it was retrieved.

With few exceptions, prior studies examining the veracity of emotional memories have tested individual participants, without considering the role of social influence. Yet when emotional events occur in daily life, social interactions are likely to be a critical part of the memory rehearsal and retrieval process. Although we might think to ourselves about mundane experiences that have occurred, we tend to reminisce and commiserate with one another about life’s highs and lows. The overarching goal of the present study was to examine whether emotionally arousing items would continue to be more accurately remembered than would neutral ones when discussion of the encoding event was encouraged, or conversely, whether this social interaction might remove or reverse the beneficial effects of emotion on memory accuracy. It is critical to address this question in order to understand whether the findings of laboratory studies of emotional memory accuracy—which typically test memory individually—have implications for the accuracy of real-world emotional events, when events are often rehearsed with others.

To date, only three published studies have examined how collaboration can affect the retrieval of emotional information. Yaron-Antar and Nachson (2006) assessed both individual and collaborative memory for the assassination of Israel’s Prime Minister Itzhak Rabin. Nominal group memory—that is, the pooled responses from participants tested individually—consisted of more details (both accurate and inaccurate) than did collaborative group memory, reflecting the collaborative inhibition effect often seen on tests of recall (Basden, Basden, Bryner, & Thomas, 1997; Weldon & Bellinger, 1997; Wright & Klumpp, 2004). Wessel, Zandstra, Hengeveld, and Moulds (2015) similarly demonstrated that individuals working as part of a triad remembered fewer correct, but also fewer incorrect, details about an emotional film clip than did individuals working alone. Harris, Barnier, Sutton, and Keil (2010) examined how collaborative discussion influenced memories for the autobiographical context and emotion of learning about the death of “The Crocodile Hunter.” They found no effect of collaboration on the autobiographical details that people reported about where and when they had learned the news, but they did find that collaboration distorted people’s memories for their experienced emotions, reducing the shock and overall emotion that participants remembered feeling. None of these studies included a neutral control event; thus, although all three demonstrate that collaborative retrieval can affect emotional memory characteristics, they leave open the question of how emotional memories fare relative to neutral ones when social interactions occur during retrieval. Moreover, these prior studies did not examine whether the positive and negative emotional memories are differently influenced by collaboration. On the one hand, there is reason to think that it may be the arousing nature of the stimuli that would influence memory (reviewed by Hamann, 2001; Yonelinas & Ritchey, 2015) and thus that the effects of collaboration would be similar for positive or negative memories. On the other hand, there is evidence that valence affects memory even when arousal is held constant; often, memory for negative stimuli are associated with greater specificity and reduced memory errors (reviewed by Kensinger, 2009), although there have been some counterexamples where negative stimuli are more prone to false memories (Brainerd et al., 2008; Porter, Taylor, & Ten Brinke, 2008). These findings raise the possibility that collaboration could have different effects on negative and positive memories.

To address these questions, we conducted two experiments, each modeled off of a different paradigm that has been used to examine the role of social interaction on nonemotional memory. This approach enabled a test of the robustness and generalizability of social influence on emotional memory for positive and negative stimuli as compared to neutral stimuli. If social influences affected memory for positive and negative stimuli similarly, this would demonstrate an effect that generalized across valences of stimuli and that would be attributable more generally to the emotional features of the stimuli. By contrast, if social influence differently impacted memories for positive and negative stimuli, this would be attributable to the valence of the memories (or to the emotional responses tied to that valence), since the positive and negative stimuli were selected to be of similarly high arousal. The first experiment used a collaborative recognition paradigm, and the second experiment used a social contagion paradigm. In brief, past studies of collaborative retrieval have demonstrated that when memory is tested by recognition, collaborative retrieval tends to produce better memory accuracy relative to recognition memory accuracy for nominal groups (Clark, Hori, Putnam, & Martin, 2000; Rajaram & Pereira-Pasarin, 2007). This enhanced accuracy can, in part, be attributed to “error pruning” (Rajaram & Pereira-Pasarin, 2007; Ross, Spencer, Blatz, & Restorick, 2008; Ross, Spencer, Linardatos, Lam, & Perunovic, 2004; see Rajaram & Pereira-Pasarin, 2010, for review) during retrieval collaboration, where feedback from group members can attenuate false memories and prevent incorrect endorsement of lure items as having been studied. This error pruning is likely to occur because not all members of the group will share the same false memory; hearing from multiple members of a group that a lure item was not studied may be sufficient to enable others to reject the item. In support of this conclusion, studies that use unrelated lures, or categorically related lures, in which the false memories would vary from participant to participant, tend to result in error pruning (e.g., Rajaram & Pereira-Pasarin, 2007; Pereira-Pasarin & Rajaram, 2011). By contrast, studies that use associative lists that all converge on a single lure item (e.g., Deese–Roediger–McDermott lists; Deese, 1959; Roediger & McDermott, 1995) often lead to collaborative enhancement of false memories (Basden, Reysen, & Basden, 2002), likely because the false memory is shared among group members (see Rajaram & Pereira-Pasarin, 2010, for discussion).

Such instances of increased false memories with retrieval collaboration highlight one of its insidious consequences. If a member of the group is providing misinformation, this information can erroneously become incorporated into other individuals’ memories. Roediger, Meade, and colleagues (Meade & Roediger, 2002; Roediger, Meade, & Bergman, 2001) developed a paradigm to reveal what they called the “social contagion of memory,” whereby erroneous information supplied by a confederate later is reported by a participant (see Gabbert, Memon, & Allan, 2003, for a related “memory conformity” paradigm). Interestingly, this erroneous information, produced by the confederate, has been shown to be endorsed at a high rate by participants even when they are given explicit warnings about social influence just prior to retrieval (Meade & Roediger, 2002).

Together, these extreme findings demonstrate that there can be both advantages and disadvantages to retrieval collaboration. Some of these differences are related to the memory reliability of the retrieval partners: When partners are monitoring for memory accuracy and are generally providing accurate information, collaboration often benefits memory accuracy. But when partners are the source of misinformation, memory errors can become inflated. The present study was designed to investigate how the accuracy of emotional memories, relative to neutral ones, would fare under both these conditions. It did so using the paradigm of Choi et al. (2013), in which participants study categorized lists of positive, negative, and neutral stimuli and later are asked to discriminate studied items from unstudied, categorically related foils. When individuals are tested alone, this paradigm has led to enhanced memory accuracy for the emotional items (Choi et al., 2013). The present study examined how collaborative retrieval would affect this pattern of results, both when the collaborators were working toward a common goal of accurate retrieval (Experiment 1) and when a confederate was intentionally introducing misinformation (Experiment 2). Together, using two thematically related paradigms, these experiments address the question of how emotion and social collaboration influence the occurrence of memory errors, thus contributing to three intersecting fields of study: false memory, emotional memory, and social memory.

## Experiment 1: Collaborative Retrieval

The first experiment used a retrieval collaboration method adapted from Rajaram and Pereira-Pasarin (2007), with the task from Choi et al. (2013). This combination allowed for the investigation of the effects of social collaboration on memory accuracy for emotional as compared to neutral information. Critically, in this paradigm, the collaborators are working together toward a common goal of accurate retrieval, and participants are explicitly told that their individual responses are of primary interest and do not need to conform to the majority response.

### Method

#### Participants

Data from 48 participants are reported. All participants were undergraduates (*M* = 19.6 years of age) at Boston College. Twenty-four of these participants were assigned to the individual test condition, and 24 were assigned to the collaborative group test condition. All were prescreened for history of psychiatric or neurological disorders and for current depression or high anxiety. An additional 12 participants were tested, but their data are not reported here because they either failed to return for the second session when memory testing occurred, were unable to form part of a collaborative triad because one or more members of their group failed to return, or, in the case of two groups, were assigned to an incorrect study list due to experimenter error. Informed consent was obtained in a manner approved by the Boston College Institutional Review Board, and participants were compensated either with course credit or at a rate of $10/hour.

Participants were scheduled in groups of four, and pseudo-randomly assigned by the experimenter to either the “individual” or “group” test categories. Participants were not told what test group they were assigned to until they returned for the test phase of the study (see Procedure section). Participants were assigned to a group together only if they did not know one another.

#### Stimuli

The stimuli and presentation scripts were those used in Choi, et al. (2013). In brief, categorized lists of stimuli were selected such that eight photo objects of categorically related stimuli were selected for a total of 45 categories (15 of negative valence, 15 of positive valence, and 15 of neutral valence). For instance, a negative category was funeral—with casket, cemetery, hearse, etc., as category members—while a positive category was wedding—with veil, flower girl, bride, etc., as category members. Photo objects were selected from those used in prior studies (Kensinger, Garoff-Eaton, & Schacter, 2007; Waring & Kensinger, 2011) and all had been normed for valence and arousal by at least 20 participants who were sampled from the same Boston-area college student population that was sampled for the present study. All negative photo objects received average ratings that were lower than 4 on a valence scale of 1 to 9 (with 1 being the most negative) and higher than 5 on an arousal scale of 1 to 9 (with 9 being the highest arousal). All positive photo objects received average ratings that were higher than 5 on valence and higher than 5 on arousal. The negative and positive photo objects were chosen so that the sets did not differ in arousal (*p* > .25) and did significantly differ in valence (*p* < .001). All of the neutral photo objects were rated between 3 and 6 on valence and lower than 5 on arousal, so that they were significantly less arousing than the positive or negative stimuli (*p* < .001) and differed in valence from both of the emotion categories (*p* < .01). The three valences of items (positive, negative, neutral) did not differ in frequency, familiarity, or imageability of their verbal referent (norms from the MRC database, all *p*s > .15). The objects from the three valence categories also did not differ in visual complexity (*F* < 1.5, *p* > .25), as determined by normative data from 20 participants. The items also did not differ in the numbers that included people, inanimate objects, animals, or landscapes across valence and categories. This matching was done by selecting the categories and photo exemplars in triplicate (e.g., there were equivalent numbers of images with people in the “in a hospital” [negative], “in a restaurant” [positive], and “in a school” [neutral] categories). Additional normative data are presented in Choi et al. (2013), who used the same stimuli and task as the present study.

Based on this stimulus selection, a valence-specific effect would be one that occurred for positive stimuli but not negative stimuli, or vice versa. A general emotion effect would be one that was shared by positive and negative stimuli as compared with neutral stimuli. Because the positive and negative stimuli were selected to differ from neutral stimuli on both valence and arousal dimensions, this effect of emotion could be tied to the valence of the stimuli, the arousal of the stimuli, or a related emotion-relevant dimension.

#### Procedure

The task was divided into two phases: a study phase and a recognition memory test phase. There was an unsupervised 48-hour delay between study and test.

#### Study phase

Participants studied the stimulus lists individually in a soundproof testing room. Participants were told that they were going to view a series of slides containing a category label at the top of the screen, with an image of an object and the object’s verbal label underneath the category label. They were told that they would have 4 seconds to decide how appropriate an exemplar the item was for the category heading—for example, if the category label was “Astronomy” and the item was a picture of Jupiter and the word *Jupiter*, they were to decide how well *Jupiter* fit in the category of “Astronomy.” Participants were instructed to make keyboard ratings on a 1 to 5 scale, with the heuristic that a 1 represented an *average fit*, a 3 represented a *rather good fit*, and a 5 represented an *excellent fit*. The scale was anchored so that a response of 1 did not represent a *bad fit* because all items were related to the category label. After a short practice, participants studied a total of 225 items, five from each of the 45 categories (15 positive, 15 negative, and 15 neutral).

The study phase was presented as an intentional learning task: participants were told that after completing the study task, they would later be tested on the items they were about to rate. They were told that testing would either occur in isolation, or in a group with two other participants, although participants were not told the retrieval condition (individual, group) to which they had been assigned. Participants were given no further information about the test phase at this point.

#### Test phase

Participants returned to the lab after 48 hours and were given instructions for either the group or individual test, based on what condition they had been assigned to after the conclusion of the first session.

#### Group instructions

All participants in the group had seen the same study list during the study phase. Participants were brought to a room and seated at a round table. Each participant was given a Macintosh laptop with a blank Microsoft Excel spreadsheet open. No other files or applications were accessible. Participants were seated such that no participant could see any other participant’s screen. A fourth “stimulus” laptop was positioned on the table to be equally visible to all participants. Participants were told that the stimulus laptop would display single words, one at a time, and participants were to deliberate as a group as to whether the item was an “old” item—seen during the previous study phase—or if it was a “new,” unstudied item. They were told to deliberate for as long as necessary but were encouraged to keep the deliberation focused on the current test item. They were told that they did not need to reach a group consensus about whether the item was old or new. They were instructed to press any key on the stimulus laptop keyboard to advance to the next trial after everyone had recorded their own response.

Participants recorded their responses to the recognition test items in a blank Microsoft Excel spreadsheet on a separate laptop. To ensure that responses were recorded for each item and that participants would not lose their place, participants were instructed to type the stimulus cue in the first column of the spreadsheet, and record their “old” or “new” response in the adjacent column. They were told that each trial should be recorded in a separate row of the spreadsheet.

Participants were presented with all 360 items: the 225 studied items and the remaining 135 items that were presented as nonstudied “lure” items. A single word appeared in the center of the screen. No images or category labels were shown during the test phase. Items that were studied or used as unstudied lures were fully counterbalanced across participants. Items in both the study and test phases were presented in an order randomly generated by E-Prime at the start of each test session.

#### Individual instructions

Participants tested individually were taken to a testing room containing a response laptop as described above, and a stimulus laptop as described above. Participants in the individual test condition were required to record their responses on a separate laptop to hold testing modality constant between the individual and group tests. The individual test proceeded identically to the group test: participants were told they could take as much time as needed to arrive at their “old” or ”new” judgment, and they recorded their responses as described previously.

#### Data analysis

Discrimination (*d*-prime) analyses were computed according to Stanislaw and Todorov (1999), with two extreme values (two false alarm rates of 0) adjusted, as in Macmillan and Kaplan (1985). Using these *d*-prime values, an analysis of variance (ANOVA) was conducted with the between-subjects factor of participant condition (collaborative, individual) and the within-subjects factor of item valence (positive, negative, neutral). Additional ANOVAs examined the effect of these factors on the hit and false alarm rates. All analyses compare the data for the 24 participants tested individually to the data for the 24 participants from the collaborative triads who also provided individual recognition responses to all items following a discussion; thus, 48 separate datasets (one per participant) were included for all analyses.

### Results

#### Discrimination (d-prime)

ANOVA revealed a main effect of valence, *F*(2, 45) = 10.81, *p* < .001, η_p_^2^ = .33. Discrimination was better for positive (*M* = 2.61, *SE* = .11) and negative items (*M* = 2.58, *SE* = .11) than for neutral items (*M* = 2.3, *SE* = .10). There was a marginal effect of participant condition, *F*(1, 46) = 2.76, *p* = .10, η_p_^2^ = .06, with better discrimination in the collaborative (*M* = 2.66, *SE* = .14) compared to the individual condition (*M* = 2.33, *SE* = .14). There also was a marginal interaction between valence and condition, *F*(2, 45) = 2.66, *p* =.08, η_p_^2^ = .11, with a larger effect of valence in the collaborative condition relative to the individual condition. Specifically, there was significantly better discrimination of both positive (*p* = .002) and negative (*p* = .001) items as compared to neutral items in the collaborative condition, and marginally better discrimination of positive (*p* = .05) and negative (*p* = .13) items as compared to neutral items in the individual condition (see Table 1).

**Table 1:** Mean (*SE*) *d*-prime values as a function of valence and participant condition in Experiment 1

Condition	Valence		
	Positive	Negative	Neutral
Individual	2.43 (.16)	2.35 (.16)	2.22 (.14)
Collaborative	2.79 (.16)	2.82 (.16)	2.37 (.14)

#### Hit rate and false alarm rate

A 2 (Participant Condition) × 3 (Valence) ANOVA conducted on the hit rates revealed a main effect of valence, *F*(2, 45) = 8.30, *p* < .005, η_p_^2^ = .27, with higher hit rates for positive (*M* = 0.86, *SE* = .01) and negative (*M* = 0.87, *SE* = .01) compared to neutral (*M* = 0.83, *SE* = .02) items. There was no effect of participant condition (*F* = 1.03), and no interaction (*F* < 1.0). A similar 2 (Participant Condition) × 3 (Valence) ANOVA conducted on the false alarm rates revealed no main effects nor interaction (all *F*s < 1.6, all *p*s > .21) (See Table 2).

**Table 2:** Mean (*SE*) hit and false alarm rates as a function of valence and participant condition in Experiment 1

Condition	Hit Valence			False Alarm Valence		
	Positive	Negative	Neutral	Positive	Negative	Neutral
Individual	.85 (.02)	.85 (.02)	.82 (.02)	.11 (.02)	.13 (.02)	.13 (.02)
Collaborative	.87 (.02)	.89 (.02)	.84 (.02)	.08 (.02)	.09 (.02)	.12 (.02)

### Discussion

The results of Experiment 1 corroborated and extended those of Choi and colleagues (2013) by demonstrating that when the associative strength of items is controlled, emotion enhances memory discriminability even when collaboration occurs during retrieval. Although the overall collaborative memory benefit seen here was perhaps weaker than in prior studies, the effect of collaboration was in the same direction as prior studies using exactly this collaborative instruction (Rajaram & Pereira-Pasarin, 2007), providing a benefit to memory discriminability. One limitation of this collaborative design relates to time-on-task differences for those assigned to the collaborative and individual conditions: Participants assigned to the group retrieval condition were likely to spend more time on each recognition trial than participants assigned to the individual condition. Importantly, while this difference could explain overall benefits in memory, it would not explain the effects of emotion. Thus, in Experiment 1, the beneficial effects of emotion remained when individuals collaborated at retrieval, and the beneficial effects of collaboration existed for emotional as well as neutral memories. For memory discriminability, there also was a marginal interaction between valence and group, whereby the emotional memory enhancement (i.e., better memory for positive and negative stimuli relative to neutral stimuli) was greater in the participants who collaborated than in the participants who were tested individually. It will be important for future research to assess whether this interaction pattern will replicate, and whether it could be strengthened under conditions that enhance the effect of collaboration—such as if participants were required to reach consensus about each item’s study history or if retrieval were tested via recall rather than recognition.

In Experiment 1, it was unlikely that all participants in a triad would share the same false memory. Thus, if one individual endorsed a lure item, it is likely that other individuals in the triad would counter that claim, indicating that a lure item was *not* studied. Even though participants in the present experiment were not required to reach a consensus as to whether an item had been studied, this type of dispute may have encouraged participants to more carefully monitor the basis for their memory decisions. Although emotion enhances memory accuracy under these circumstances, this raises the question: How will emotion affect memory accuracy when the participant is in a context in which a collaborator is a frequent source of misinformation and there is no opportunity for adjudication? This was the question that Experiment 2 was designed to answer.

## Experiment 2: Social Contagion During Practice

The design of Experiment 2 was adapted from Roediger et al. (2001), to examine what they termed “the social contagion of memory.” In brief, individual participants performed the same encoding and recognition task as in Experiment 1. Prior to completing the recognition task, half of the participants performed a category-cued collaborative recall task with a confederate and the other half performed the recall task on their own. The confederate introduced a number of lure items in response to the category cues. The study was, therefore, designed to investigate how this form of social interaction—shown by Roediger and colleagues (Roediger et al., 2001; Meade & Roediger, 2002) to increase the participants’ endorsement of the confederate-supplied lure items—would affect memory accuracy for emotional and neutral information.

### Method

#### Participants

Data are reported from 48 participants (20 men, 28 women), recruited by online ads and bulletin board ads targeting undergraduate and graduate students in the greater Boston area. Participants were 20 years of age on average (range from 18 to 28). Due to experimental error, an additional five participants were assigned to incorrect study lists, thereby disrupting the counterbalancing assignment; their data are excluded from analysis. An additional six participants completed the study phase but failed to return for the second session when memory testing occurred. Of the 48 participants whose data were analyzed, 24 were assigned to a social contagion condition (11 men) and 24 to a control condition (nine men).

#### Stimuli

The stimuli and presentation scripts were those used in Choi et al. (2013) and were identical to those used in Experiment 1.

#### Procedure

The procedure was divided into three parts: an encoding phase, a recall phase that took place either with a confederate or alone (depending upon the participants’ condition), and a recognition phase.

#### Encoding phase

The encoding phase exactly followed the methods described in Choi and colleagues (2013), and the methods were identical to those used in Experiment 1.

Five items from each category were presented as study items whereas three items were reserved to serve as nonstudied items for the assessment of false memories. Eight study lists counterbalanced across participants the categorized stimuli that were studied versus that were reserved as lures on the recognition test. All participants were told that their memory would be tested for the items, although they were given no further information about the subsequent memory test.

#### Recall (practice) phase

After an approximately 48-hour delay (required to be between 46 and 50 hours), participants returned to the laboratory. They were shown category labels (e.g., “funeral”) and were asked to recall items from the list (e.g., hearse, casket). Participants in the control condition performed this task alone. Participants in the contagion condition were told that they were going to work together with another person, using a video call to be able to see and hear the other person. Participants were familiar with the video call format, and many of them spontaneously commented that they use video calls to correspond with their family and friends in other locations. Unbeknownst to the participant,^1^ this other person was a confederate of the research team, who had been given a specific set of words to supply for each category (The video call format was used so that the confederate could consult a master list that indicated which words she should recall for each category.) The participant took turns recalling words with the confederate (e.g., the participant would recall a word, then the confederate, then the participant), alternating across trials whether the participant or the confederate supplied the first word. Across all categories, the confederate supplied 15 lures of each valence and 15 hits of each valence. For any given category, the number of lures supplied by the confederate ranged from 0 to 2; this variability was intentional, to minimize the likelihood that participants would become suspicious of the confederate. The particular words supplied by the confederate varied across the eight study lists, but this breakdown of lures and hits was constant across all lists.

After this recall phase, the participant was given a 30-minute break. Participants were offered an opportunity to briefly leave the laboratory to stretch their legs, and they performed Sudoku puzzles during the remainder of the break.

#### Recognition phase

Participants were instructed that they would be working alone to complete a final memory test. They were presented with individual words on the screen and were asked to judge whether each word had been studied during the encoding session two days earlier by making an appropriate button press to indicate “old” or “new.” This recognition test was self-paced.

#### Data analysis

Discrimination (*d*-prime) analyses were computed according to Stanislaw and Todorov (1999), with two extreme values (a hit rate of 1 and a false alarm rate of 0) adjusted as in Macmillan and Kaplan (1985). Using these *d*-prime values, an analysis of variance (ANOVA) was conducted with the between-subjects factor of participant condition (contagion, control) and the within-subjects factor of item valence (positive, negative, neutral). Additional ANOVAs examined the effect of these factors on the hit and false alarm rates. For participants in the contagion condition, we additionally divided the lure items into those that had been supplied by the confederate and those that had not been supplied by the confederate and examined the influence of this factor on false endorsements.

### Results

#### Discrimination (*d*-prime)

ANOVA revealed a main effect of valence on discrimination ability, *F*(2, 45) = 12.18, *p* < .001, η_p_^2^ = .36, with better discrimination for positive (*M* = 1.4, *SE* = .09) and negative items (*M* = 1.4, *SE* = .09) than for neutral items (*M* = 1.2, *SE* = .08). There was no main effect of participant condition and no interaction (both *F*s < 1). Thus, having interacted with the confederate did not reduce the overall ability to discriminate studied from nonstudied items in the subsequent memory task (see Table 3 for all *d*-prime values).

**Table 3:** Mean (*SE*) *d*-prime and Hit rates as a function of valence and participant condition in Experiment 2

Group	*d* -prime values		
	Positive	Negative	Neutral
Control	2.43 (.15)	2.35 (.14)	2.22 (.13)
Contagion	2.79 (.17)	2.82 (.17)	2.37 (.16)
	Hit rates		
Control	.75 (.02)	.77 (.02)	.74 (.02)
Contagion	.80 (.02)	.82 (.02)	.81 (.02)

#### Hits

The ANOVA revealed a main effect of valence, *F*(2, 45) = 4.49, *p* = .02, η_p_^2^ = 0.17, with better memory for negative words (.80) than for positive (.78) or neutral (.77) words. There also was a main effect of condition, *F*(1, 46) = 4.31, *p* = .04, η_p_^2^ = .09, with higher hit rates in the contagion condition (.81) than in the control condition (.75). There was no significant interaction between valence and condition, *F* < 1, *p* = .51 (see Table 3 for all hit rates).

#### False alarms

The false alarm rates are reported in Table 4. When the false alarm rate to all lures was analyzed, the ANOVA revealed a main effect of valence, *F*(2, 45) = 11.26, *p* < .001, η_p_^2^ = .33, with higher false alarm rates for neutral lures (.35) than for positive (.29) or negative (.31) lures. There was no effect of condition, *F*(1, 46) = 1.45, *p* = .23, and no interaction, *F* < 1, *p* = .84. Thus, overall false alarm rates were not inflated by the social contagion manipulation. However, effects of the social contagion manipulation were revealed when we compared the false alarm rates in the confederate group and the control group on the subset of items that were supplied by the confederate, or that would have been supplied by the confederate had the control participant been in that condition.^2^ This ANOVA showed a main effect of valence, *F*(2, 45) = 6.22, *p* = .004, η_p_^2^ = .22, with higher false alarms to neutral lures (.50) than to positive (.41) or negative (.43) lures. There was a main effect of condition, *F*(1, 46) = 20.65, *p* < .001, η_p_^2^ = .31, with participants in the confederate condition having higher false alarm rates (.56) than those in the control condition (.32). There was no interaction between valence and condition, *F*(2, 45) = 2.15, *p* = .13, η_p_^2^ = .09.

**Table 4:** Mean (*SE*) False Alarm rates as a function of valence and participant condition in Experiment 2

Condition	Valence		
	Positive	Negative	Neutral
Contagion supplied	.53 (.05)	.54 (.05)	.66 (.04)
Contagion not supplied	.22 (.03)	.23 (.03)	.23 (.03)
Contagion overall	.32 (.03)	.34 (.03)	.37 (.03)
Control	.26 (.03)	.29 (.03)	.33 (.03)

**Fig. 1:**
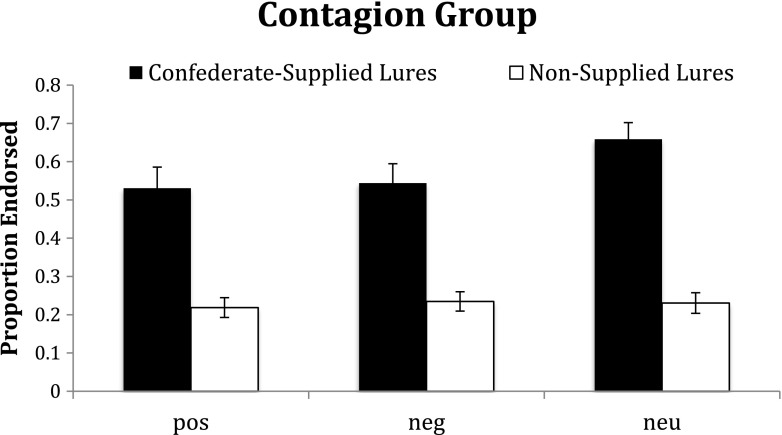
For participants in the contagion group, lure words supplied by the confederate (black bars) were more likely to be falsely recalled than lure words not supplied by the confederate (white bars), particularly if the lures were neutral

We also examined the effect of social contagion only within the contagion group, comparing the false alarm rates for lures supplied by the confederate and for lures not supplied by the confederate. This ANOVA revealed a main effect of valence, *F*(2, 22) = 6.04, *p* = .008, η_p_^2^ = .35, with higher false alarm rates for neutral lures (.44) than for positive (.37) or negative (.38) lures. This ANOVA also revealed an effect of supplied items, *F*(1, 23) = 78.33, *p* < .001, η_p_^2^ = .77, with higher false alarm rates to the supplied lures (.56) than to the nonsupplied lures (.23). Critically, these main effects were qualified by a significant interaction, *F*(2, 22) = 4.51, *p* = .02, η_p_^2^ = .29: Although the effect of the supplied items was strong for all valences (all *p*s < .001), neutral items showed a larger effect (*t* = 9.95) than negative (*t* = 6.60) or positive items (*t* = 5.66; see Fig. 1).

### Discussion

Experiment 2 confirmed the insidious consequences of the social contagion of memory, with very high endorsement of lures that had been supplied by the confederate. The results further demonstrate that emotional memories are not immune to this type of influence; although the effect is lessened for emotional lures, participants still endorse the emotional lures supplied by the confederate at a high rate (over 50 % of the time). Yet, even under these conditions, in which memory distortion is rampant, emotional information is more accurately remembered than neutral information. These results are reminiscent of those reported by C. Brown and Schaefer (2010); using a social conformity paradigm, they demonstrated that postevent misinformation had a lesser influence on recognition memory for emotional (positive or negative) pictures than for neutral pictures. Together, these findings emphasize that emotion can buffer against memory distortion, even when that distortion is elicited via misinformation provided in a social interaction.

## General Discussion

Across both experiments, an emotional enhancement of memory was present, with better discrimination of emotional targets and lures as compared to neutral targets and lures. This beneficial effect of emotion on memory existed both when social partners were working together to achieve accurate memory decisions (see Experiment 1) and when a social partner was the frequent provider of misinformation (see Experiment 2). Next, we discuss the relevance of these findings for the understanding of emotional memory, social memory, and false memory.

### Contributions to emotional memory

The results corroborate those of Choi and colleagues (2013), demonstrating that when the associative strength of target and lure items is comparable across valence categories, emotion enhances memory discriminability. The present study provides the first evidence that these beneficial effects of emotion can be maintained when collaboration occurs during retrieval, and when a practice partner has introduced misinformation. These findings suggest a resiliency of emotional memory to misinformation effects and suggest that emotion may aide in the accurate retention of information in real-life contexts, in which emotional events are often the topic of social discussion.

The results further confirm that emotional memories are not immune to distortion (e.g., Pesta et al., 2001) while also revealing that emotional memories can be less susceptible to distortion across a range of social influences. Why might this be? Some aspects of source memory are enhanced by emotion, including the ability to remember whether something was imagined or visually presented (Kensinger, O’Brien, Swanberg, Garoff-Eaton, & Schacter, 2008). In the social contagion paradigm, it is possible that emotional items are somewhat protected from the misinformation provided because participants are better at remembering which items were visually presented at study and which were supplied by the confederate. In both experiments, it is possible that participants are more likely to invoke a distinctiveness heuristic (reviewed by Schacter, Israel, & Racine, 1999) when collaborators supply one of the lure items. Participants may disregard the veracity of the collaborator’s memory for an emotional lure, believing that they would have remembered the emotional item had it been presented. These are suppositions; further research will be needed to determine the mechanisms that lead to the increased accuracy for emotional memories demonstrated in both experiments.

Interestingly, in the present study, the effects of emotion extended to items of positive or negative valence. Because these emotional items were intentionally chosen to be of higher arousal than the neutral items, these effects cannot be pinpointed as effects of valence or effects of arousal. That is, the effects could either be tied to the emotional items being of nonneutral valence or to the emotional items being of higher arousal. The results clearly demonstrate that both positive and negative emotion can enhance memory discriminability, and this enhanced discriminability can remain strong even when the emotional events are discussed with others.

It should be noted that in both experiments, the recognition task was completed 48 hours after the study phase. Although Choi et al. (2013) revealed beneficial effects of emotion on memory accuracy after both a short (30-minute) and a longer (48-hour) delay, the effects of emotion on memory can become exaggerated over delays. Indeed, there has been much recent discussion as to the reason for the shallower decay rate for emotional as compared to neutral information (e.g., Yonelinas & Ritchey, 2015), including the potential importance of sleep for the preservation of emotional information (e.g., Bennion, Payne & Kensinger, 2015). Thus, the relatively long delay length in the present study—although chosen primarily for practical reasons (to avoid ceiling effects in memory and for convenience of scheduling the participants)—may also have intensified our ability to uncover the beneficial effects of emotion on memory.

### Contributions to social memory

A growing literature has demonstrated that social interactions can lead individuals to be less likely to endorse nonstudied lures (e.g., error pruning in collaborative retrieval; reviewed by Rajaram & Pereira-Pasarin, 2010) or to be more likely to endorse nonstudied lures (e.g., social contagion effects; Roediger et al., 2001). The present study demonstrates that this general conclusion holds for emotional items as well as for neutral items. Consistent with the patterns for neutral information, the effects of social interaction on emotional memory accuracy were in opposite directions in the two experiments. In Experiment 1, when members of the collaborative triad were unlikely to share the same false memory, and when they were given an opportunity to discuss any discrepancies in memory, the false alarm rates for emotional lures were numerically (3 %–4 %) lower in the collaborative retrieval condition as compared to the individual retrieval condition. By contrast, in Experiment 2, when the confederate frequently supplied misinformation and the paradigm did not enable the participant to confront the confederate about the veracity of this information, the false alarm rates were approximately twice as high for emotional lures supplied by the confederate in Experiment 2 as compared to control items. Thus, as with memory for more mundane experiences, whether social interaction helps or hurts the accuracy of emotional memory depends on the memory accuracy of the retrieval partner and whether there is an opportunity for adjudication when disagreements in memory arise. Importantly, by including both neutral and emotional items within the same study, the present results also revealed that, as compared to memory for neutral items, emotional memories are more accurate even when social interactions encourage the endorsement of erroneous information. The social contagion effect was significantly reduced by emotion—the effect for neutral items was one-third more than the effect for emotional items—demonstrating that participants may be less influenced by the reports of others when retrieving details of past emotional events.

The results of the social contagion experiment further reveal that interactions with a confederate do not influence overall discrimination ability. Participants who interacted with a confederate did not differ from controls in their overall discrimination rates. Rather, interaction with the confederate led to an increase in the endorsement of items that were supplied by the confederate and to a decrease in endorsement of items that were not supplied by the confederate. This latter finding is unexpected, and future research will be needed to determine its reliability and to investigate its underlying mechanisms. We suggest two possibilities. First, this effect may reflect part-set cuing, whereby the generation of some lures by the confederate may make other lures less accessible in memory, leading the nonsupplied lures to seem less familiar when presented on the subsequent recognition test. Second, the contagion group has two potential sources of errors: confederate-supplied lures and self-generated lures. It is possible that the confederate-supplied lures take a preferential focus, leading to lower endorsement of self-generated lures than is seen in the control participants, whose only source of errors are these self-generated lures.

### Contributions to false memory

The results clearly demonstrate that rates of false memories can be influenced by social collaboration, by emotion, and by the interaction of these factors. Moreover, the results of Experiment 2 reveal that specific false memories can be created via social interaction even when the overall ability to discriminate items is not affected. The fact that those participants in Experiment 2 who recalled items with a confederate later endorsed over half of the nonstudied items supplied by the confederate—and double the percentage of neutral items endorsed by participants in a control condition—underscores the potential for social interactions to robustly affect the accuracy of memory reports. Together, these results emphasize that to understand the veracity of memory, the social and emotional context of the memories must be considered.

### Conclusions

In daily life, emotional events are often discussed with others. The present study was the first to examine the effect of these social interactions on the veracity of emotional memories as compared to neutral ones. Across two very different collaborative paradigms, emotional memories were more accurate than neutral memories. These results reveal, for the first time, that emotional events can be more accurately remembered than neutral ones across a range of social retrieval contexts. This result brings us one step closer to understanding the veracity of emotional memories in real-world contexts, where social rehearsal and reminiscing are likely to be key features.

## Footnotes

1 During the debriefing procedure, participants were informed about the presence of the confederate and were probed as to whether they had surmised this role for the other person; no participant reported being aware that their partner was a confederate.

2 When an ANOVA was conducted for the nonsupplied lures (and the corresponding lures had the control participants been in the confederate group), the analysis revealed a main effect of valence, *F*(2, 45) = 5.10, *p* = .01, η_p_^2^ = .19, with higher false alarms to neutral lures (.27) than to positive (.23) or negative (.25) lures. There was no effect of condition, *F*(1, 46) = 2.06, *p* = .15, η_p_^2^ = .04. There was an interaction between valence and condition, *F*(2, 45) = 3.48, *p* = .04, η_p_^2^ = .09, with a lower false recognition rate for positive (*p* < .001) and negative (*p* = .04) items compared to neutral items in the control group but not in the confederate group (all *p*s > .5).
